# Response to correspondence article on the research protocol titled *Towards Health Equity and Transformative Action on tribal health (THETA) study* *to describe, explain and act on tribal health inequities in India: A health systems research study protocol*

**DOI:** 10.12688/wellcomeopenres.19190.1

**Published:** 2023-04-04

**Authors:** Prashanth N Srinivas, Tanya Seshadri, Nandini Velho, Giridhar R Babu, C Madegowda, Yogish Channabasappa, Sumanth Mallikarjuna Majigi, Deepa Bhat

**Affiliations:** 1 Field station BR Hills, Institute of Public Health Bengaluru, Chamarajanagar, Karnataka, 571441, India; 2 Independent Researcher, Panjim, Goa, 403001, India; 3 Indian Institute of Public Health, Bangalore, Karnataka, 560023, India; 4 Jilla Budakattu Girijana Abhivruddhi Sangha, Chamarajanagar, Karnataka, 571441, India; 5 Department of Community Medicine, Mysore Medical College and Research Institute, Mysore, Karnataka, 570001, India; 6 Department of Anatomy, JSS Medical College, Mysore, Karnataka, 570015, India

**Keywords:** Tribal health, Transformative action

## Abstract

In this correspondence, we, co-authors and collaborators involved in the *Towards Health Equity and Transformative Action on tribal health (THETA) study* respond to a recent article published in Wellcome Open Research titled *Correspondence article on the research protocol titled ‘Towards Health Equity and Transformative Action on tribal health (THETA) study to describe, explain and act on tribal health inequities in India: A health systems research study protocol’ published in Wellcome Open Research in December 2019 *In the first part, we provide overall clarifications on the THETA study and in the second part respond to specific comments by the authors of the aforementioned correspondence.

As the research team and author group and collaborators of the *Towards Health Equity and Transformative Action on Tribal Health* (THETA), we were happy to see the protocol paper ^1^ (hereafter referred to as THETA protocol paper) receive attention from Kumar *et al.* ^2^. We were also concerned that some misconceptions about our study procedures appear in the article, which we seek to clarify. These details may have been missed from our protocol article and supporting documents or may have been unclear.

To start with, we shall provide some context to this study and core elements of its multi-method design. The overall approach used is that of health policy and systems research ^3^, where the choice of methods is contingent upon the nature of research questions which in turn is conceptualised as a way of advancing scientific inquiry for the purposes of improving people’s health and addressing inequities ^4^.

As mentioned in the proposal, THETA was based on work that began in 2014 under *Participation for Local Action* research project in close collaboration and with the participation of researchers from the *Soliga* Adivasi community as partners in the research process ^5^. Based on the experience of participatory action research under this project, and based on the findings of inequalities at a finer-scale than is assessed by currently available nation-wide demographic and health surveillance survey data ^6^, we expanded our footprint to include sites in Kerala, Madhya Pradesh and Arunachal Pradesh. The objectives of the THETA study were covered in figure 1 of the THETA protocol paper ^1^ and reproduced below:

1. Objective 1 (Pattern): To describe and analyse the nature and extent of health inequalities among forest-dwelling tribal communities in three major tribal regions;
  a. Tribal communities have poor health and nutrition status indicators when compared to non-tribal people in the same area
  b. Remoteness alone does not explain this difference in health and nutrition status indicators
2. Objective 2 (Process): To explain the underlying reasons for health inequity among tribal communities through a contextualized and empirically validated theory.
3. Objective 3 (Action): To design and pilot an intervention to address health inequity in tribal communities.

As clarified in the THETA protocol paper, the intervention design was expected to be “based on a (program) theory that draws from the refined theories from studying the processes for inequities, and hence the intervention is also an opportunity to validate/refine the program theories on processes driving inequities” ^1^.

The project was part of a DBT/Wellcome Trust India Alliance Intermediate Fellowship in Clinical/Public Health Research, awarded to the first author (2017–2022). The project is now completed and while some of the research outputs are already published (cited below), others are in preparation. In addition, there have also been public engagement including a planetary health interpretation center established in Pakke Tiger Reserve in collaboration with the forest department and community members with a three short film series showcasing the co-production process ^7, 8^ and policy engagement outputs including an innovative tribal healthcare navigation program that was announced by the department of tribal welfare, Government of Karnataka based on Adivasi patient healthcare experiences shared with policymakers in course of policy engagement undertaken in THETA project. Several community workshops and several dissemination meetings have been held for instance, a dissemination meeting held in August 2022, where the Chamarajanagar Institute of Medical Sciences took the leadership in hosting a tribal health cell. The establishment of tribal health cells was based on continuous policy engagement led by THETA project team and the Sangha of the Adivasi communities, based on the inputs to improve research participation in tribal health by community medicine departments ^9^.

THETA project involved extensive community involvement and engagement especially in objectives 2 and 3 (it was only objective 1 which was conducted in multiple sites across India, while objective 2 and 3 was restricted to southern Karnataka site as already mentioned in the THETA protocol paper). This work in southern Karnataka is long-term work in partnership with the *Sangha* (collective community-based organisation of the *Soliga* Adivasi people) and with local NGOs; it is based on ongoing long-term collaborative MoUs signed between research team with the *Sangha,* local NGOs and district and state government health and tribal welfare departments.

Kumar *et al.* raise five points that we feel require further clarification from our side.

First, they raise a critical point about comparison of variables related to tribal and non-tribal community living in the same geographical area. Kumar *et al.* contend in their commentary that such a comparison "...may not give insights into the causal factors of inequities as they are more dependent on the socio-cultural contexts of communities rather than on sharing the same geographies." In our project, the core hypothesis indeed was to test this assumption because the patterns of inequities among tribal and non-tribal communities living in the same geography is not the same across India. For instance, being an Adivasi in southern Karnataka or in northern Kerala may be different from the tribal-non-tribal situation in central or north-east India. In order to understand these fine-scale geo-spatial inequities, our hypothesis aimed to characterise the nature of inequities at these smaller scales. A preliminary analysis showing the diverse nature of these inequities across states and sites is published ^6^. In our ongoing analysis, we aim to further characterise the nature of tribal identity and how it operates geo-spatially in and around multiple protected areas through the dataset and we hope to be able to share this upon completion of the analysis later this year. We hope that such data shall show the *pattern* of differences within and across the common *Scheduled Tribe (ST)* label and help us identify entry-points for locally relevant socio-cultural inquiry. One of the ways we aim to do this is by examining the nature of geographic location versus social isolation both of which together contribute to the socio-geographical construct of *remoteness* in the context of Adivasi communities. This is because being geographically distant from villages and cities need not automatically result in social isolation as is the case with several other communities which live far away but are not as socially isolated as some ST communities are. Kumar *et al.* also suggest to "...study the health differences within the sub-groups of indigenous community in the same geographies." We concur. It is indeed with this intention that we have proposed the fine-scale sampling at protected area level across different regions of the country as described in the THETA protocol paper ^1^. And our upcoming analysis is guided by this very argument in line with some ongoing collaborative work that emerged while conducting the THETA study on malnutrition differences within and across tribal communities that was recently published from northern Kerala led by our Kerala study site collaborator ^10, 11^. From the THETA dataset too, malnutrition patterns that report within tribe differentials and across tribe differentials in malnutrition patterns will be reported on. 

Second, Kumar *et al.* assert that "the collection of biological samples, genetic testing and saving samples in a biorepository seem to be inconsistent with the research title". While this is indeed true, the title of transformative action on tribal health emerges not from objective 1 (which is quantitatively focused and biomedical in its orientation) but from objective 3 which draws from narrative data, leverages existing social science theories and uses participatory action research methods. The transformative action element is built upon the intent in THETA project to work closely with one of the sites in southern Karnataka (as stated in the THETA protocol paper) through a long-term partnership that the study team had established in 2014 with the *Zilla Budakattu Girijana Abhivruddhi Sangha*, a community-based organisation of the Soliga Adivasi community with whom we have a long-term MoU and have been core members of our previous and ongoing research work. Indeed, one of the co-authors and collaborators on the THETA study is a research scholar from the Soliga community and currently the secretary of the Sangha. The work done in objective 3 has already led to outputs that aim to improve our understanding of the practice of participatory action research with Adivasi communities. In our previous publications, we examine what CBOs could do for more effective researcher-CBO partnerships ^12^, critically reflect upon understanding of how inclusiveness in health systems research priority-setting is affected when community organizations lead the process ^13^ and based on the ongoing work, examine how to overcome barriers to such collaborations between researchers and communities ^14^. At our survey sites in northern Kerala and Arunachal Pradesh, our engagement continues through researchers and collaborations we established and the hope indeed is to engage in researcher-led participatory action in these sites too. For instance, a foundation for this has been laid in our site in western Arunachal Pradesh through local collaborations with the forest department, researchers and community members through the establishment of a planetary health/onehealth interpretation center ^7^. A detailed documentation of the co-production process applied in establishing this center is ongoing while we worked with local filmmakers to produce short videos documenting the process ^8^.

Third, Kumar *et al.* question the use of recorded verbal consent in the presence of a witness as a method of obtaining consent from tribal communities. At each site we used a recorded video in local tribal language and recorded by a local tribal woman as a means of communicating the information including purpose of the study, risks and benefits. Based on piloting this approach in Kerala, Arunachal Pradesh and Karnataka sites, we found this to be an effective means of obtaining consent as it was not perceived to be threatening (needing to give written consent on paper forms) and was more culturally acceptable. Based on this, we informed our ethics committee on the possible risks associated with this approach taking into account also the possible exclusion of various participants if mandatory written consent was imposed on each participant. Given that we were working with local collaborators, community-based organisations and gatekeepers in all study sites (except the Central Indian site), we concluded that the implementation of a recorded verbal consent in the presence of a witness could be an effective way of obtaining informed consent. Indeed as pointed out by Kumar *et al.*, this is exceptional taking into account the need to ensure participation of communities living in remote settings, and is NOT to be considered routine as clarified the national guidelines on ethics in human participant research published by the Indian Council of Medical Research in 2017 (the same year when THETA protocol was finalised). We argue that the method of consent has to be done on a case-to-case basis taking into account a reflexive discussion between the research team and participant community representatives as well as the nature of the research inquiry.

Fourth, regarding the collection of genetic material and storage in a bio-repository, this was done only for the southern Karnataka study site and not done at any other site. This decision was taken because in the southern Karnataka site, there was a robust procedure and a system in place to ensure full safety to participants and also most importantly, to be able to provide ancillary care wherever the results of the blood sample showed any signs of ill-health. This was undertaken as a sub-study nested within THETA and led by one of the co-authors (SMM) and funded via a grant to the department of community medicine from their multi-disciplinary research unit at Mysore Medical College. This sub-study component received additional ethics clearance from the ethics committee of Mysore Medical College. This study added the biological component over and above the THETA study’s socio-demographic component. A detailed paper reporting on the findings is in preparation. The purpose for isolation of genetic material was for confirmation of sickle cell disease that is well documented in Adivasi communities but poorly researched, as shown by our health systems scoping review under THETA project ^15^. The argument for storage of some samples was in case there was a need for any new test or tool for sickle cell disease in the future given the possible benefits to the participants. This component was taken up through a new collaboration established under THETA project with DB at JSS Medical College, Mysore where work towards genetic analysis of hemoglobinopathies had already begun under an ICMR Haemoglobinopathies National Task Force Project. As mentioned clearly in the protocol already, this component could have proceeded only if consent would have been re-established for any genetic testing beyond the purposes that we collected it for (genetic testing for sickle cell disease). Given the diagnostic and therapeutic benefit to the participating individuals, and additional benefit of seeking preventive genetic counselling in case family members tested positive, we argue for collection of a blood sample. A total of 88 individuals with no prior knowledge of their sickle cell status tested positive and they are all in the process of being enrolled into the treatment program that is now being implemented with the government medical college and our collaborators. A manuscript reporting the details of the results from sickle cell disease testing is in preparation and the test results and subsequent ancillary care to participating individuals is now completed. We are glad to share that we were able to build upon the initial work done in THETA study to eventually propose a hemoglobinopathy registry to be maintained at the district health department and government medical college in Chamarajanagar district, for which preparations are now ongoing. 

Fifth, Kumar *et al.* point out the possible need to have submitted this protocol to the health ministry's screening committee, which according to the official website "will review the research projects involving international collaboration/funding in health research" ^16^. This proposal was entirely funded via the DBT/Wellcome Trust India Alliance, an Indian funding agency as part of a competitive research fellowship awarded within the country. No external funding was received for accomplishing any of the components in this protocol. 

We are grateful to receive scrutiny and attention from eminent scholars in the field and invite more discussion and debate on the critical topics raised. As we move ahead with establishing a Center for Training Research and Innovation in Tribal Health (through a centre grant awarded by the DBT/Wellcome Trust India Alliance), now with collaborations with government and private medical college, public health research institutes, state and district level health and tribal welfare departments and with the Adivasi Sanghas, these elements will be underscored in our training, teaching and writing.
