# Supplementary material for: Response to correspondence article on the research protocol titled Towards Health Equity and Transformative Action on tribal health (THETA) study to describe, explain and act on tribal health inequities in India: A health systems research study protocol
Source: Wellcome Open Res. 2023 Apr 4;8:155. [Version 1] doi: 10.12688/wellcomeopenres.19190.1 (PMC10521096; doi:10.12688/wellcomeopenres.19190.1)
Supplement: Peer review history

# Reviewer report: Reviewer response for version 1

**DOI:** 10.21956/wellcomeopenres.21271.r64878

**Published:** 2023-09-11

**Authors:** Manu Raj Mathur

**Affiliations:** 1 Queen Mary University of London, London, England, UK

The article is in response to a correspondence that was submitted in response to the THETA study protocol by the authors. There were a number of questions raised based on the technical, ethical and administrative aspects of the research. I read the original article as well as the correspondence and I think the authors have answered all the questions very comprehensively and with confidence that is a reflection of true work on the field.

 My only concern is the response to the question on HMSC clearance. I think the authors should have been more detailed in answering this question. It is not only about the funding of projects but also involves any foreign collaboration associated with the publication of manuscript. One sentence on how foreign collaborating authors will be accommodated in manuscript needs to be highlighted. Also, all the international collaborations are needed to be listed to maintain transparency. Being a part of multiple ethical committees, this is becoming increasingly important and although the work is funded by India Alliance, disclosure of such information will only be facilitative of future academic dissemination.

Are arguments sufficiently supported by evidence from the published literature or by new data and results?

Yes

Is the conclusion balanced and justified on the basis of the presented arguments?

Yes

Is the rationale for commenting on the previous publication clearly described?

Yes

Are any opinions stated well-argued, clear and cogent?

Yes

Reviewer Expertise:

Health policy and systems research, oral health promotion and non-communicable diseases

I confirm that I have read this submission and believe that I have an appropriate level of expertise to confirm that it is of an acceptable scientific standard.

# Reviewer report: Reviewer response for version 1

**DOI:** 10.21956/wellcomeopenres.21271.r60857

**Published:** 2023-07-24

**Authors:** Arima Mishra

**Affiliations:** 1 Azim Premji University, Bengaluru, Karnataka, India

I read the authors’ responses to the comments by Kumar *et al., *(2023) raised on the THETA protocol published earlier by the team.

 The authors have systematically and clearly responded to each of the five points/concerns raised by Kumar *et al.* They have provided additional context, clarifications as well as drawn on relevant publications from their ongoing work (the published THETA protocol is part of this larger work) to substantiate their responses. The responses indeed have not only clarified the important points raised by Kumar *et al.,* but do enrich discussion on tribal health and inequities and process of co-production of knowledge including ethical concerns.

Are arguments sufficiently supported by evidence from the published literature or by new data and results?

Yes

Is the conclusion balanced and justified on the basis of the presented arguments?

Yes

Is the rationale for commenting on the previous publication clearly described?

Yes

Are any opinions stated well-argued, clear and cogent?

Yes

Reviewer Expertise:

Tribal health, health inequalities, qualitative research, health systems policies and practices

I confirm that I have read this submission and believe that I have an appropriate level of expertise to confirm that it is of an acceptable scientific standard.
